# 氟达拉滨和环磷酰胺联合利妥昔单抗（FCR方案）一线治疗慢性淋巴细胞白血病43例临床分析

**DOI:** 10.3760/cma.j.issn.0253-2727.2021.07.003

**Published:** 2021-07

**Authors:** 婷玉 王, 树华 易, 轶 王, 瑞 吕, 齐 王, 书会 邓, 伟薇 隋, 明伟 傅, 文阳 黄, 薇 刘, 刚 安, 耀中 赵, 录贵 邱

**Affiliations:** 中国医学科学院血液病医院（中国医学科学院血液学研究所），实验血液学国家重点实验室，国家血液系统疾病临床医学研究中心，天津 300020 State Key Laboratory of Experimental Hematology, National Clinical Research Center for Blood Diseases, Institute of Hematology & Blood Diseases Hospital, Chinese Academy of Medical Sciences & Peking Union Medical College, Tianjin 300020, China

**Keywords:** 白血病，淋巴细胞，慢性, 氟达拉滨, 环磷酰胺, 利妥昔单抗, 治疗结果, Leukemia, lymphocytic, chronic, Fludarabine, Cyclophosphamide, Rituximab, Treatment outcome

## Abstract

**目的:**

探讨FCR方案（氟达拉滨+环磷酰胺+利妥昔单抗）一线治疗慢性淋巴细胞白血病（CLL）的疗效。

**方法:**

回顾性分析2004年5月至2017年12月一线应用FCR方案治疗的43例CLL患者的临床资料。

**结果:**

①43例CLL患者中，男31例，女12例，接受FCR方案治疗时中位年龄58（36～72）岁；8例患者伴B症状，外周血中位淋巴细胞计数26（3～550）×10^9^/L，IGHV基因未突变62.1％（18/29），P53基因缺失14.0％（6/43），RB1基因缺失18.6％（8/43），12号染色体三体占25.6％（11/33），ATM基因缺失16.7％（7/42）。全部患者FCR方案中位疗程数为4（2～6）个。②全部43例患者的总体反应率（ORR）为88.4％（38/43），完全缓解（CR）20例（46.5％），部分缓解（PR）18例（41.9％），疾病稳定（SD）4例（9.3％），疾病进展（PD）1例（2.3％）；7例（16.3％）患者获得微小残留病（MRD）阴性。③中位随访51（6～167）个月，中位无进展生存（PFS）时间为67（29～105）个月，中位总生存（OS）时间未达到，5年PFS率为（62.1±8.6）％，10年PFS率为（31.0±14.3）％，5年OS率为（70.5±8.3）％，10年OS率为（51.3±13.8）％。疗程数<4为影响OS的不良预后因素，P53基因缺失、疗程数<4为影响PFS的不良预后因素（*P*<0.001），且在多因素分析中仍具有预后意义［P53基因缺失：*HR*＝7.65（95％*CI* 1.74～33.60），*P*＝0.007；疗程数<4：*HR*＝3.75（95％*CI* 1.19～11.80），*P*＝0.025］。④18例（41.9％）患者于化疗后发生2～3级感染，19例（44.2％）发生3～4级血液学不良反应，1例（2.3％）患者发生肿瘤溶解综合征，所有不良反应经对症处理均恢复。

**结论:**

FCR方案一线治疗CLL的治疗反应及远期生存较理想，不良反应可接受。

## Abstract (en)

**Objective:**

To investigate the efficacy of fludarabine and cyclophosphamide combined with rituximab（FCR）in previously untreated patients with chronic lymphocytic leukemia（CLL）.

**Methods:**

The clinical data of 43 enrolled patients from May 2004 to December 2017 were analyzed the efficacy and survival results.

**Results:**

A total of 43 patients with 31 males and 12 females, and the median age was 58 years old（range 36 to72）before treatment. There were 8 patients with symptom B. The median number of peripheral blood lymphocyte was 26（3–550）×10^9^/L. IGHV unmutated was detected in 62.1％（18/29）patients, P53 deletion in 14％（6/43）patients, RB1 deletion in 18.6％（8/43）patients, Trisomy 12 in 25.6％（11/33）patients, ATM deletion in 16.7％（7/42）patients, respectively. The median number of treatment courses administered was 4（range 2–6）. Twenty patients obtained CR（46.5％）, 18 patients obtained PR, 4 patients were SD, 1 patient was PD. The overall response rate（ORR）was 88.37％. Seven patients obtained MRD negative. After the median follow-up time of 51（6–167）months, median PFS was 67（29–105）months, median OS was not reach, 5-year PFS was（62.1±8.6）％, 10-year PFS was（31±14.3）％, 5-year OS was（70.5±8.3）％, and 10-year OS was（51.3±13.8）％. Less than 4 courses predicted adverse OS（*P*<0.05）. P53 deletion and less than 4 courses were associated with poor PFS（*P*<0.001），and the prognostic value still remained after multivariate analysis［*HR*＝7.65（95％*CI* 1.74–33.60）, *P*＝0.007; *HR*＝3.75（95％ *CI* 1.19–11.80）, *P*＝0.025］. Eighteen patients（41.9％）appeared grade 2–3 infection after chemotherapy, and 19 patients（44.2％）appeared grade 3–4 hematological adverse reactions. One patient（2.3％）was developed tumor lysis syndrome. All adverse reactions were controlled or recovered spontaneously.

**Conclusion:**

Previously untreated CLL patients treated with FCR had a high response rate and good survival rate, which is an important treatment choice for fit patients.

慢性淋巴细胞白血病（CLL）是一种共表达CD5和CD23、形态单一的成熟小B淋巴细胞惰性肿瘤，中位发病年龄72岁，西方国家的年发病率为4.75/10万，亚洲国家（地区）的年发病率为1.06/10万[1]。CLL的治疗历经了细胞毒化疗（包含苯丁酸氮芥、氟达拉滨、环磷酰胺）、FCR方案（氟达拉滨+环磷酰胺+利妥昔单抗）和苯达莫司汀联合利妥昔单抗的免疫化疗阶段，目前已步入小分子靶向药物的新药时代。在治疗方式的发展过程中，CLL的疗效逐步提高，生存时间进一步延长。免疫化疗在小分子靶向药物出现之前在CLL一线治疗中起着举足轻重的作用，CLL8临床研究[2]奠定了FCR方案作为年轻具有治疗指征的体能状态良好CLL患者的一线治疗推荐，即使是在新药时代，对于年轻、体能状态良好、无TP53基因异常、IGHV基因突变型的CLL患者仍是一线治疗选择。本研究对我中心以FCR方案一线治疗的43例CLL患者进行回顾性分析，旨在探讨FCR方案一线治疗CLL的疗效。

## 病例与方法

1. 病例：本研究纳入2004年5月至2017年12月期间在我院淋巴瘤中心接受FCR方案一线治疗的43例CLL患者。根据2001年版WHO淋巴瘤分类标准以及国际慢性淋巴细胞白血病工作组（iwCLL）2008指南[3]进行诊断，所有患者均进行骨髓或外周血流式细胞术免疫分型、骨髓病理形态学及免疫组化检查。

2. 治疗方案：氟达拉滨25 mg·m^−2^·d^−1^，第1～3天静脉滴注；环磷酰胺250 mg·m^−2^·d^−1^，第1～3天静脉滴注；利妥昔单抗375 mg/m^2^，氟达拉滨和环磷酰胺前1天静脉滴注；每疗程28 d。

3. 疗效评价：参照iwCLL2008指南[3]进行疗效评价，分为完全缓解（CR）、部分缓解（PR）、疾病稳定（SD）、疾病进展（PD）、复发。

4. 随访：通过电话联系方式进行随访。随访截止时间为2020年6月1日。总生存（OS）期定义为诊断之日至患者死亡或随访截止日期的间隔时间。无进展生存（PFS）期定义为患者开始接受治疗之日至任何原因所致复发、死亡或随访截止的间隔时间。

5. 统计学处理：统计学处理：采用SPSS软件进行数据分析。采用Kaplan-Meier生存法绘制生存曲线。不同预后因素的生存差异比较采用Log-rank检验。采用Cox回归模型进行PFS、OS的多因素分析并进行似然比检验。双侧*P*<0.05为差异有统计学意义。

## 结果

1. 患者临床特征：43例患者中，男31例，女12例，开始FCR方案治疗时中位年龄为58（36～72）岁。43例患者中，脾大25例（轻度脾大5例，中度脾大12例，巨脾8例），行脾切除术者1例。初诊血常规（中位数）：WBC 30.1（3.7～561.1）×10^9^/L，淋巴细胞计数26（3～550）×10^9^/L，HGB 110（51～168）g/L，PLT 150（41～323）× 10^9^/L。Binet分期：A期4例（9.3％），B期23例（53.5％），C期16例（37.2％）；Rai分期：0期1例（2.3％），Ⅰ期8例（18.6％），Ⅱ期13例（30.2％），Ⅲ期14例（32.6％），Ⅳ期7例（16.3％）。IGHV基因突变型占37.9％（11/29），IGHV基因未突变型占62.1％（18/29）。荧光原位杂交技术（FISH）检测P53基因缺失6例（14.0％），RB1基因缺失8例（18.6％），12号染色体三体占25.6％（11/33），ATM基因缺失16.7％（7/42）。13例患者中位外周血CLL细胞比例为0.89（0.46～0.97），34例患者中位骨髓CLL细胞比例为0.650（0.080～0.960）。8例患者伴有B症状（发热、盗汗、体重减轻）。

2. 治疗反应：43例CLL患者完成FCR方案化疗的中位疗程数为4（2～6）个，其中31例（72.1％）疗程数≥4个，12例（27.9％）完成6个疗程。疗程数<6个患者中有12例因化疗后骨髓明显抑制而减少1～2疗程，其他19例患者因个人原因中断治疗。43例患者中CR 20例（46.5％），PR 18例（41.9％），SD 4例，PD 1例，总体反应率（ORR）为88.4％（38/43）；完成2疗程（43例）、完成4疗程（34例）患者的CR率分别为16.3％（7/43）、47.1％（16/34）（*P*＝0.004）；完成6疗程（12例）患者的CR率为85.7％（12/14），与完成4疗程组CR率差异有统计学意义（*P*＝0.040）。7例（16.3％）患者（均为CR）获得MRD阴性，CR患者的MRD阴性率为35％（7/30），完成4、6疗程患者的骨髓MRD转阴率分别为17.6％（6/34）、35.7％（5/14）（*P*＝0.001）。

3. 随访和预后：43例患者中位随访时间为51（6～167）个月，18例发生复发/进展，其中4例发生Richter转化，2例发生第二肿瘤（Ph^+^急性淋巴细胞白血病、骨髓转移腺癌各1例），死亡12例。中位PFS期为67（29～105）个月，中位OS未达到。5年PFS率为（62.1±8.6）％，10年PFS率为（31.0±14.3）％，5年OS率为（70.5±8.3）％，10年OS率为（51.3±13.8）％。生存曲线见图1。

**图1:**
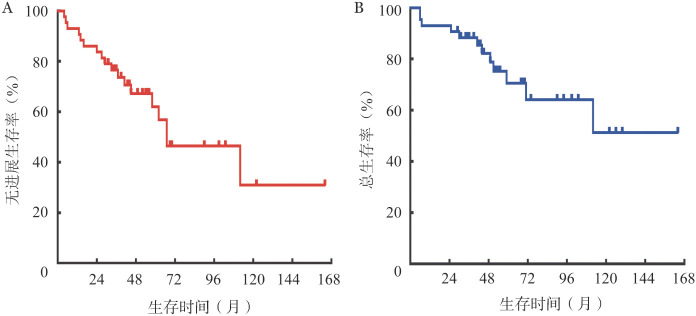
43例接受FCR方案一线治疗慢性淋巴细胞白血病患者的生存曲线 FCR方案：氟达拉滨+环磷酰胺+利妥昔单抗；A：无进展生存曲线；B：总生存曲线

对Rai分期、Binet分期、血清LDH水平、β_2_微球蛋白、IGHV基因未突变、RB1基因缺失、ATM基因缺失、12号染色体三体、P53基因缺失、完成4疗程化疗、完成5疗程化疗、疗程数<4、诱导化疗结束骨髓MRD阴性进行单因素预后因素分析，结果显示疗程数<4为影响OS的不良预后因素，P53基因缺失、疗程数<4为影响PFS的不良预后因素（详见表1），且在多因素分析中仍具有预后意义［P53基因缺失：*HR*＝7.65（95％*CI* 1.74～33.60），*P*＝0.007；疗程数<4：*HR*＝3.75（95％ *CI* 1.19～11.80），*P*＝0.025］。共有29例患者进行IGHV高突变分析，其中IGHV突变型11例（1例同时合并17p−），IGHV未突变型18例。IGHV突变型患者的5年PFS率、OS率分别为（70.1±14.7）％、（79.5±13.1）％，IGHV未突变型患者的5年PFS率、OS率分别为（36.5±14.7）％、（48.1±14.5）％，差异均无统计学意义（*χ*^2^＝3.181，*P*＝0.075；*χ*^2^＝0.703，*P*＝0.402）（生存曲线见图2）。因回顾性资料数据欠缺，仅对19例患者进行了CLL-IPI评分，其中低危患者2例、中危患者6例、高危患者7例、极高危患者3例，5年OS率分别为100.0％、（83.3±15.2）％、（44.4±22.2）％、0（*χ*^2^＝15.35，*P*＝0.002）。细胞遗传学无异常（14例）、伴P53基因缺失（6例）、伴ATM基因缺失（6例）、单独RB1基因缺失（5例）、单独12号染色体三体（3例）、单独IGH重排（6例）患者的5年OS率分别为（67.3±16.0）％、（50.0±20.4）％、（83.3±15.2）％、（80.0±17.9）％、100.0％、（53.3±24.8）％（*χ*^2^＝5.274，*P*＝0.383）。

**表1:** 43例接受FCR方案一线治疗慢性淋巴细胞白血病患者生存相关变量单因素Kaplan-Meier分析结果

因素	例数	5年PFS			5年OS		
		率（％）	*χ*^2^值	*P*值	率（％）	*χ*^2^值	*P*值
Rai分期			0.040	0.841			
2～4	34	58.2±10.0			62.3±10.1		
0～1	9	77.8±13.9			100.0±0.0		
Binet分期			0.067	0.795		0.283	0.595
B+C	39	60.5±9.3			66.9±9.2		
A	4	75.0±21.7			100.0±0.0		
LDH			1.700	0.192		0.656	0.418
≥247U/L	16	48.6±12.8			67.5±12.1		
<247U/L	23	76.5±9.5			74.8±11.2		
β_2_微球蛋白			0.646	0.422		0.046	0.831
>3.5mg/L	16	40.1±15.2			47.3±14.9		
≤3.5mg/L	11	81.8±11.6			100.0±0.0		
IGHV基因突变状态			3.181	0.074		0.703	0.402
未突变	18	36.5±14.7			48.1±14.5		
突变	11	70.1±14.7			79.5±13.1		
RB1基因缺失			0.628	0.428		0.775	0.379
阳性	8	72.9±16.5			87.5±11.7		
阴性	35	60.3±9.6			67.5±9.4		
ATM基因缺失			1.585	0.208		2.592	0.107
阳性	7	47.6±22.5			71.4±17.1		
阴性	35	66.3±9.3			73.8±9.0		
12号染色体三体			0.007	0.934		0.011	0.916
阳性	11	54.5±15.0			72.7±13.4		
阴性	22	65.0±12.2			71.2±12.6		
P53基因缺失			29.414	<0.001		2.801	0.094
阳性	6	16.7±15.2			50.5±20.4		
阴性	37	70.0±9.0			74.1±8.8		
疗程数比较1			0.252	0.616		0.748	0.387
疗程数4	11	68.2±15.8			72.9±16.5		
疗程数5～6	20	83.6±8.8			94.4±5.4		
疗程数比较2			16.007	<0.001		9.930	0.002
疗程数<4	12	16.7±14.4			29.2±16.2		
疗程数4～6	31	78.7±7.9			87.1±7.0		
化疗后骨髓MRD			0.001	0.980		0.087	0.768
阳性	18	58.5±12.2			72.4±12.2		
阴性	6	62.5±21.3			83.3±15.2		

注：FCR方案：氟达拉滨+环磷酰胺+利妥昔单抗；MRD：微小残留病；PFS：无进展生存；OS：总生存

**图2:**
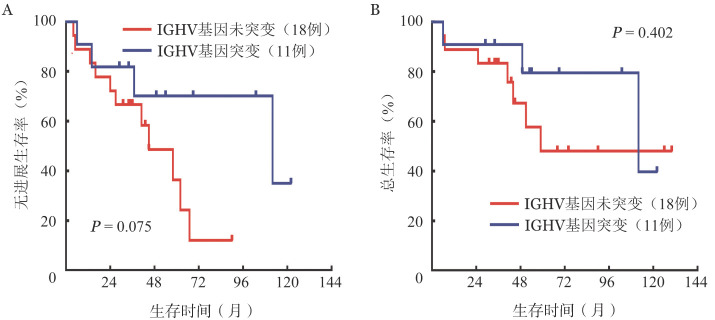
IGHV基因突变、未突变慢性淋巴细胞白血病患者FCR方案一线治疗后生存曲线 FCR方案：氟达拉滨+环磷酰胺+利妥昔单抗；A：无进展生存曲线；B：总生存曲线

4. 治疗相关不良反应：43例患者中有18例（41.9％）化疗后发生2～3级感染，包括细菌感染18例、真菌感染5例，中性粒细胞减少性发热3例、病毒感染2例；共19例（44.2％）患者发生3～4级血液学不良反应，其中粒细胞减少12例（3级7例、4级5例），血小板减少4例（3级3例、4级1例），贫血3例（3级2例、4级1例）。1例患者发生肿瘤溶解综合征。所有不良反应经对症处理后均恢复。

## 讨论

CLL作为一种惰性的小B细胞淋巴瘤，有1/3的CLL患者诊断后终生不需要接受治疗，1/3的患者在诊断后经一段时间观察后开始治疗，还有1/3的患者诊断时具备治疗指征需要立即接受治疗[4]。作为一种惰性肿瘤，CLL患者的治疗目标在于控制肿瘤、缓解疾病、延长生存、提高生存质量。在免疫化疗时代，FCR方案是年轻、体能良好CLL患者的首选推荐方案。在CLL8临床研究[5]中FCR组ORR为90％，CR率为44％，中位随访5.9年，FCR组中位PFS 56.8月，中位OS未达到，获得了在免疫化疗时代较之于其他CLL一线治疗方案[6]–[9]更佳的治疗反应率和生存结果。本组CLL患者获得与CLL8临床研究[5]相近的治疗反应率（ORR 88.37％，CR 46.5％）和生存结果（中位随访51个月，中位PFS超过5年，中位OS未达到，10年OS率51.3％）。我们的研究中包含Richter转化的第2肿瘤发生率13.9（6/43），与CLL8临床研究[5]中13.1％的发生率接近。可以看到FCR方案在真实世界中针对年轻耐受性好的初诊CLL患者也能到达很好的疗效。但因FCR方案存在较明显的骨髓抑制，导致血液学毒性发生率较高，在CLL8临床研究[2]中FCR组3～4级血液学毒性的发生率为56％（中性粒细胞减少34％，血小板减少7％，贫血5％）。3～4级感染发生率为25％（非特指性感染21％，细菌感染3％，真菌感染<1％）。本研究中44.2％的患者发生3～4级血液学不良反应，32.6％的患者发生3级感染，无4级感染发生，治疗相关毒性与CLL8临床研究相当。

FCR方案对CLL患者有很好的疗效，对12号染色体三体的患者效果尤其好，在CLL8研究[4]和本研究中都达到了接近临床治愈的疗效。但FCR方案不能克服P53基因缺失的不良预后因素，在CLL8临床研究[5]中FCR方案组P53基因缺失亚组中位生存仅11.3月，IGHV突变型伴有P53缺失的患者中位OS期也仅14.3个月。我们的研究中P53基因缺失的患者PFS期为13个月，明显短于无P53基因缺失的患者（PFS 112个月，*P*＝0.000），两者的OS期分别为112、25个月（*P*＝0.094），在OS中的差异未达到统计学意义，可能与本研究总的样本数量偏少有关。不同细胞遗传学分组的OS比较差异也未达到统计学意义，同样也可能存在上述原因。在免疫化疗时代，作为预后极高危的P53缺失的CLL患者缺乏有效的治疗手段，中位生存时间仅不足2年，进入新药时代后，以Bruton酪氨酸激酶（BTK）抑制剂为代表的小分子靶向药物显著改善了伴P53基因缺失CLL患者的生存，而作为第一代BTK抑制剂伊布替尼治疗伴有P53基因异常初诊CLL患者获得6年PFS率61％和OS率79％的疗效[10]，复发/难治患者也能获得7年PFS率17％和OS率39％的疗效[11]，较免疫化疗效果有明显提高。更新的BTK抑制剂如Acalabrutinib、BCL-2抑制剂维奈克拉以及PI3K抑制剂也使TP53基因异常CLL患者的生存得到明显改善[12]。最新版NCCN指南也推荐各类小分子靶向药物作为TP53基因异常CLL患者的治疗选择[13]。

IGHV基因未突变型的CLL患者较突变型的患者预后差，FCR方案不能克服其预后不良因素[2]。在我们的患者进行预后因素分析中，IGHV基因未突变状态的患者在PFS和OS上均显示出生存更差的趋势，但因检测的病例数有限，差异未达到统计学意义。进入新药时代后，小分子靶向药物伊布替尼可以克服IGHV基因未突变的不良预后因素[14]。在针对接受伊布替尼治疗的CLL患者进行的探索研究中，影响CLL患者的不良预后因素为TP53基因异常、曾经接受过治疗、β_2_微球蛋白>4 mg/L，IGHV基因未突变状态不再包括在新的预后体系中[15]，但仍需要更多的研究来论证。在E1912临床研究[16]中，对于总体患者IR组在PFS和OS上均优于FCR组，但亚组分析的结果显示对于IGHV基因突变型的CLL患者IR组和FCR组两者的生存是相当的。即便是在新药时代仍然可以对年轻的CLL患者选择性应用FCR方案治疗。

CLL-IPI[17]是新药时代前针对CLL的预后评分系统，包含TP53基因状态、IGHV基因突变状态、β_2_微球蛋白、临床分期及年龄5个指标，根据评分将患者分为低危组、中危组、高危组和极高危组，预测不同的生存结果。我们的研究中进行了CLL-IPI评分的19例患者5年OS差异显著，低危组为100％，极高危组为0，体现了接受FCR方案化疗的CLL患者可应用CLL-IPI很好地区分预后。

由于FCR方案较强的血液学毒性并导致感染并发症发生率增高，导致治疗的耐受性降低，在CLL8临床研究[2]中FCR组中位完成5.2疗程。本组患者中位完成疗程数4疗程，不足6疗程的患者中12例是因化疗后骨髓明显抑制而减少1～2个疗程，经预后因素分析，减少1～2疗程的化疗未缩短OS和PFS。完成4～6疗程化疗的患者可获得较高的CR率，完成6疗程化疗的患者骨髓MRD转阴率更高。

本研究及E1912临床研究[15]结果均显示年轻（<65或70岁）、IGHV基因突变状态、不伴有17p−的CLL患者一线应用FCR方案可获得较好的生存。因FCR方案的血液学毒性和感染并发症，可根据患者的耐受性适当减少1～2个疗程而不影响总体生存。因其为固定疗程治疗，患者在4～6个疗程数结束达到PR或CR疗效后停药观察，定期随访监测。如果出现复发并具备治疗指证后，可根据复发前缓解持续的时间以及有无克隆演变（出现新的高危细胞遗传学及分子生物学异常）选择BTK抑制剂治疗或再次行免疫化疗。

我们应用FCR方案治疗初治CLL患者达到了与CLL8临床研究中FCR组相近的疗效，显示FCR方案在免疫化疗时代是年轻、耐受性良好CLL患者的一线治疗选择，进入小分子靶向药物的新药时代，FCR方案作为固定有限周期的治疗方案对于年轻、体能状态良好、无TP53基因异常、IGHV基因突变型的CLL患者仍然可以作为首选治疗推荐。
